# Mercury Toxicity Mimicking Pheochromocytoma

**DOI:** 10.1210/jcemcr/luaf152

**Published:** 2025-08-01

**Authors:** Aisha Ansar, Kyle McNerney, Bess Adkins Marshall, Carleigh Hebbard, Sarah Berg, Michael Mullins

**Affiliations:** Department of Pediatrics, Washington University in St. Louis, St. Louis, MO 63110, USA; Department of Pediatrics, Washington University in St. Louis, St. Louis, MO 63110, USA; Department of Pediatrics, Washington University in St. Louis, St. Louis, MO 63110, USA; Department of Emergency Medicine, Washington University in St. Louis, St. Louis, MO 63110, USA; Department of Emergency Medicine, Washington University in St. Louis, St. Louis, MO 63110, USA; Department of Emergency Medicine, Washington University in St. Louis, St. Louis, MO 63110, USA

**Keywords:** mercury, pheochromocytoma, toxicity, Ayurvedic supplements, case report

## Abstract

Mercury intoxication can cause hypertension in children and mimic pheochromocytoma. We present an 8-year-old girl who presented with night sweats, intractable myalgias, hypertension, and tachycardia. Her electrocardiogram showed sinus tachycardia, and her echocardiogram was normal. Her electrolytes, creatinine, and urinalysis were normal. A thyroid function panel showed normal TSH with slightly elevated free T4. Computed tomography of the chest, abdomen, and pelvis was negative. She was discharged with antihypertensives but did not show significant improvement in her symptoms. Further workup showed normal renin levels and elevated 24-hour urinary norepinephrine, epinephrine, metanephrines, and normetanephrines. Pheochromocytoma was suspected.

On readmission and additional questioning, we learned that the patient was prescribed Ayurevedic supplements, which she had been taking for 3 months. These were discontinued 2 weeks prior to presentation when the patient first became symptomatic. Her blood and urine mercury levels were elevated. She was started on oral chelation therapy with succimer. Her hypertension was treated with doxazosin. One week after the initiation of therapy, her symptoms improved, and she was normotensive. Mercury toxicity produces a state of catecholamine excess resembling pheochromocytoma. Any diagnostic evaluation for suspected pheochromocytoma in a child should include testing and inquiry about possible sources of mercury.

## Introduction

Pheochromocytoma is a catecholamine-producing tumor arising from either the adrenal medulla or a sympathetic ganglion. It results in acute onset of hypertension, tachycardia, profuse sweating, irritability, insomnia, and lethargy. Mercury intoxication, although rare, is known to mimic the symptoms of pheochromocytoma. We report a case of mercury toxicity resembling pheochromocytoma.

## Case Presentation

An 8-year-old girl presented to the emergency department (ED) with progressive night sweats and intractable and severe migratory myalgias. Her vital signs included heart rate (HR) 125 beats per minute, blood pressure (BP) 146/116 mmHg (99th percentile for age), respiratory rate of 22 breaths per minute with 97% oxygen saturation on room air and temperature 36.3 °C. She was in her usual state of health until 2 weeks before presentation when she started to have worsening intermittent pains in her chest, arms, back, and knees. She also experienced restlessness and an inability to sleep.

**Figure 1.**
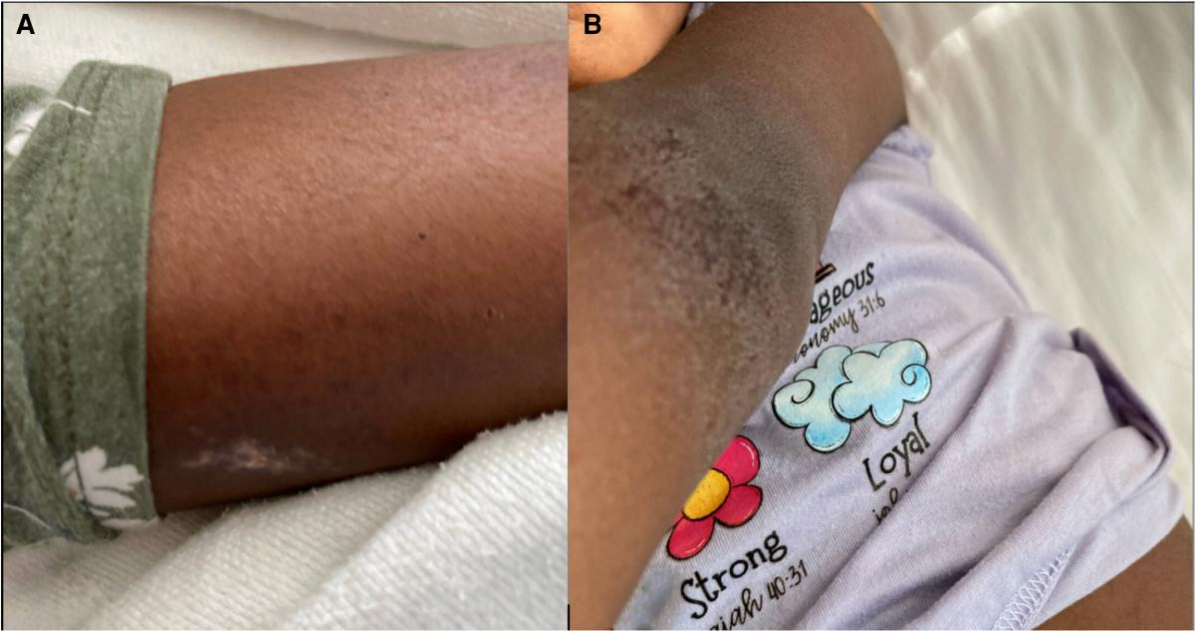
(A) Rash on forearm. (B) Rash on elbow.

In the ED, her blood pressure and heart rate remained consistently elevated. She was anxious and irritable and seemed uncomfortable. Her skin was dry and rough with a scaly, eczematous rash on the arms and legs (Fig. 1). She had persistently elevated BP in the upper and lower extremities. Her systolic BP ranged from 120 to 148 mmHg with diastolic BP ranging from 72 to 90 mmHg. An electrocardiogram showed sinus tachycardia with no conduction abnormalities. Transthoracic echocardiogram was normal. A fundoscopic examination was also normal. The patient was admitted for further workup and treatment. Based on a presumptive diagnosis of pheochromocytoma, she received oral antihypertensive medications. However, despite treatment with antihypertensives and anxiolytics, her symptoms failed to improve.

**Figure 2.**
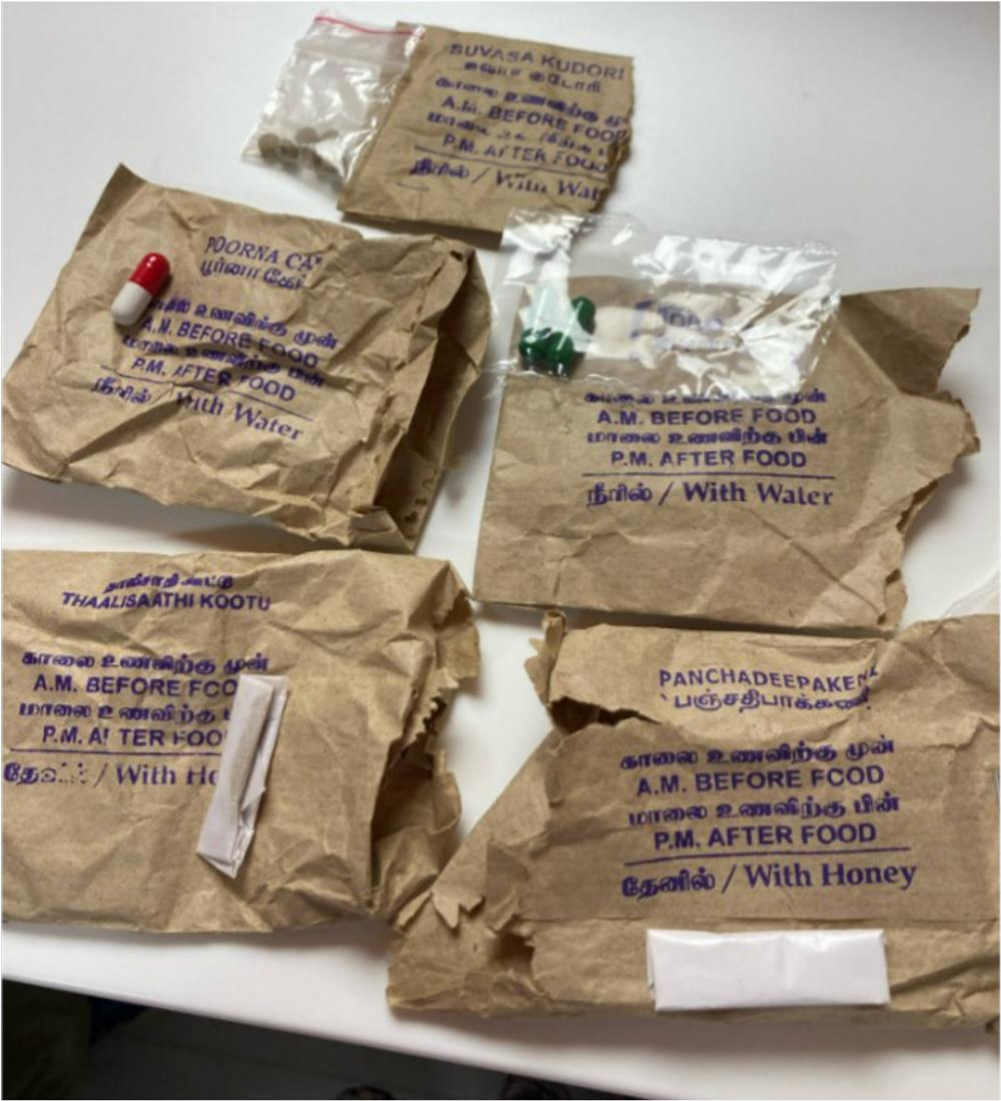
Samples of the Ayurvedic medications provided by the family.

Further questioning on her second hospitalization revealed that the patient was taking Ayurvedic supplements prepared by hand by medicinal personnel in India 3 months prior to presentation. These were stopped a few weeks prior to her presentation, when she first became symptomatic. The supplements included Ayurvedic ingredients including Idijazthi, Paneer poo, Suvasa Kadori, Elam, and Pooruacap (Fig. 2). Her parents took these supplements as well.

## Diagnostic Assessment

Her electrolytes, creatinine, and urinalysis were all normal and remained so on serial evaluations. Urine drug screen was negative. Thyroid function panel showed normal TSH with slightly elevated free T4 2.28 ng/dL (SI: 29.37 pmol/L) (reference range 0.9-1.7 ng/dL [SI: 11.6-21.9 pmol/L]) but a normal free T3 (at 3.9 pg/mL [SI: 5.0 pmol/L]) (reference range 2.0-4.4 pg/mL [SI: 3.1-6.8 pmol/L]). Free T4 measured by equilibrium dialysis/tandem mass spectrometry was normal (at 1.8 ng/dL [SI: 23.2 pmol/L]) (reference range 0.8-2.0 ng/dL [SI: 10.3-25.7 pmol/L]).

Computed tomography of the chest, abdomen, and pelvis was negative and did not show any adrenal masses or other lesions.

Plasma renin activity was 4.6 ng/mL/hour (SI: 5.93 pmol/L/hour), supine, (age-adjusted normal 0.9-2.0 ng/mL/hour [SI: 1.16-2.58 pmol/L/hour]), and her plasma aldosterone concentration <4.0 ng/dL (SI: 51.48 pmol/L) (normal <40 ng/dL while supine [SI: 514.8 pmol/L]) was normal. Her 24-hour urinary norepinephrine was 90 μg/24 hours (normal 13 to 65 μg/24 hours), epinephrine 37 μg/24 hours (normal 0.2 to10 μg/24 hours), urine metanephrines 195 μg/24 hours (normal 18-144 μg/24 hours), and urine normetanephrines were elevated at 305 μg/24 hours (normal 29 to 145 μg/24 hours).

A blood mercury concentration obtained 13 days after the first admission was elevated at 11 ng/mL (SI: 14.17 pmol/L) (normal <10 ng/mL [SI: 12.9 pmol/L]), and urinary mercury excretion was 67 μg/24 hours (normal <10 μg/24 hours).

## Treatment

Based upon an initial diagnosis of pheochromocytoma, she started treatment with antihypertensive medications with 12.5 mg atenolol daily. On hospital day 3, her dose increased to 25 mg daily with the addition of 1 mg doxazosin nightly on hospital day 5. She was sent home on these medications but had no improvement in her HR and little improvement in her BP. Outpatient treatment with acetaminophen, methocarbamol (both given for pain), and alprazolam (0.25 mg at bedtime as needed for sleep) failed to improve her symptoms.

She returned to the ED 5 days later with continued irritability, extremity pain, and rash with hypertension and sinus tachycardia. After suspicion of mercury poisoning and confirmation with blood and urine results, she began oral chelation therapy with succimer (INN: 2,3-dimercaptosuccinic acid) 10 mg/kg thrice a day for 5 days, then 10 mg/kg twice a day for 10 days. This resulted in gradual resolution of her symptoms of irritability, myalgia, rash, and insomnia. Her doxazosin dose was increased to 2 mg daily with improvement in her BP. Her BP was 106/64 mmHg at her follow-up nephrology clinic appointment 3 weeks after hospital discharge, and doxazosin was discontinued. Her symptoms were fully resolved by the time of her toxicology clinic appointment 2 further weeks later, and her vital signs included BP 101/61 mmHg and HR 102 beats per minute. The toxicology service recommended no repeat testing for mercury.

## Outcome and Follow-up

One week after the initiation of chelation therapy, her symptoms began to improve, and she remained normotensive. Her acrodynia (painful extremities) and irritability resolved. She was seen by toxicology, who recommended follow-up if any new concerns arise. She returned to school and resumed normal activities without long-term medications.

Her mother, who had also been using the same Ayurvedic medication, subsequently sought care for headaches, bilateral leg pain, and new-onset hypertension. Her pain failed to respond to acetaminophen, ibuprofen, naproxen, and prednisone. She sought testing from an outside reference laboratory 2 months after her daughter's first hospital admission. Her random blood mercury concentration was 10 ng/mL (upper limit of the normal range). Her random urine mercury concentration then was 25 ug/L (normal 0-19 ug/L) with a 24-hour urine mercury excretion of 60 μg/24 hours (normal 0-20 μg/24 hours). Her ratio of mercury to creatinine was 74 μg/g (normal 0-5 μg/g). She began chelation treatment with oral succimer. Her pain had improved before her follow-up appointment 2 months later.

## Discussion

Lead, mercury, and arsenic have been detected in a substantial proportion of Indian-manufactured traditional Ayurvedic medicines. Metals may be present due to the practice of rasa shastra (combining herbs with metals, minerals, and gems) [1].

Mercury poisoning can cause multiple-organ dysfunction and can have endocrinological findings, including effects on sodium homeostasis, thyroid function, and disordered catecholamine metabolism [2]. Similar cases have occurred worldwide [2-9] among children with confirmed exposure to elemental mercury.

Common clinical features include acrodynia, hypertension, tachycardia, mental status changes, and dermatological abnormalities. Key laboratory findings include elevated catecholamine concentrations and elevated mercury concentrations in plasma or urine. Although the clinical findings resemble pheochromocytoma, further testing excludes it. Symptoms improve with chelation therapy [2-9]. Previous cases have most often involved dermal and inhalational exposures to metallic mercury [9]. Our patient's exposure involved inorganic mercury salts ingested in Ayurvedic medication, resulting in similar hypertension, acrodynia, and skin rashes.

**Figure 3.**
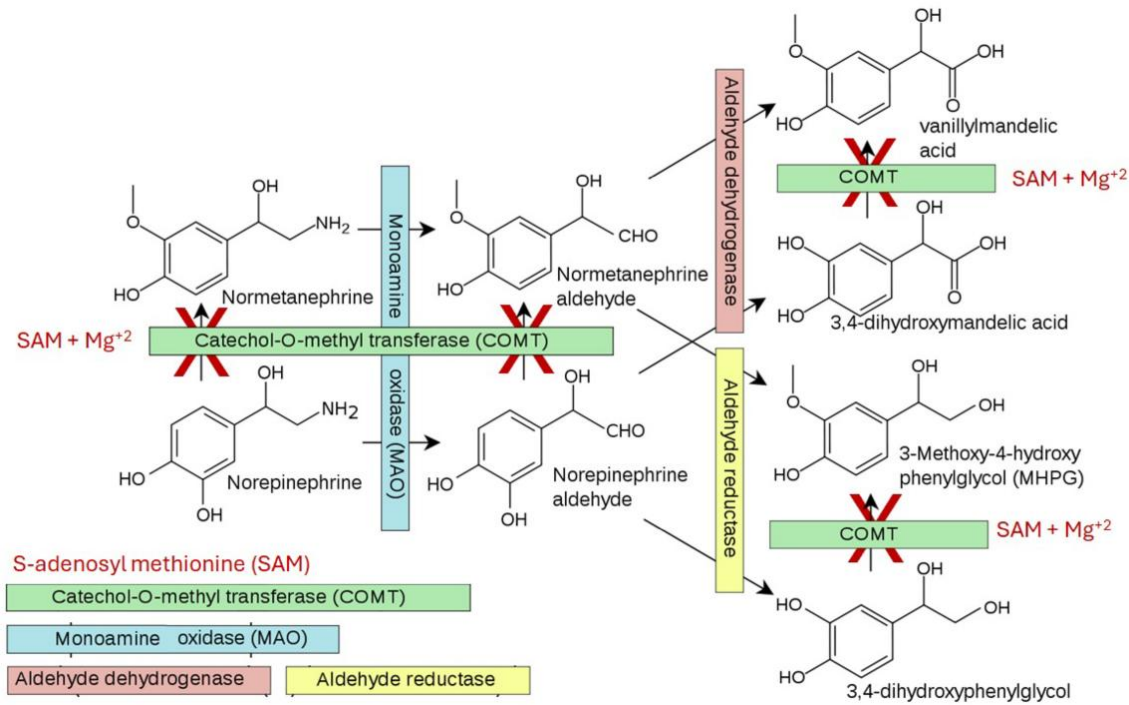
Degradation pathways for norepinephrine. SAM and Mg^+2^ are necessary cofactors for COMT. Hg^+2^ displaces Mg^+2^ and disrupts COMT function. (Adapted from reference [10] under the Creative Commons CC0 1.0.). Abbreviations: COMT, catechol O-methyltransferase; Hg^+2^, mercury; Mg^+2^, magnesium; SAM, S-adenosyl methionine.

Mercury causes elevation of catecholamine concentrations as it interferes with the catabolism of catecholamines by catecholamine-O-methyltransferase (COMT) (Fig. 3). COMT requires inactivating the coenzyme S-adenosyl methionine (SAM) and magnesium. Hg^+2^ displaces magnesium from SAM and interferes with SAM binding to COMT [10-12]. The resulting COMT inhibition leads to the accumulation of norepinephrine, epinephrine, and dopamine.

The optimal medication for BP control remains unclear. β-adrenoreceptor blockers may reduce tachycardia, but atenolol had little effect on BP in this case. Doxazosin, an α-1 adrenoreceptor blocker, is an option, but it was not highly effective in this case. α-methyldopa reduces catecholamine synthesis, but published experience with it is lacking. Clonidine (α-2 adrenoreceptor agonist) also is a potential choice.

In summary, our patient had mercury exposure that produced clinical signs and symptoms that were suggestive of pheochromocytoma. Chelation therapy led to full recovery. We suggest that evaluation of a child with suspected pheochromocytoma should include testing for mercury.

## Learning Points

- Pheochromocytoma is a rare diagnosis. Evaluation of possible pheochromocytoma should include early testing for mercury toxicity to avoid extensive diagnostic testing.
- Acrodynia, hypertension, and tachycardia associated with mercury poisoning are reversible with chelation therapy.
- A careful history of intake of supplements can be important in cases with unusual symptoms.

## Contributors

All authors made individual contributions to authorship. A.A., K.M., B.A.M., C.H., S.B., and M.E.M. were involved in the diagnosis and management of this patient. A.A., K.M., and B.A.M. were part of the endocrine team taking care of the patient. C.H., S.B., and M.E.M. were part of the toxicology team taking care of the patient. A.A. and M.E.M. were responsible for the writing and subsequent revisions. All authors reviewed and approved the final draft.

## Data Availability

Data sharing is not applicable to this article as no datasets were generated or analyzed during the current study.
